# Coping difficulties after inpatient hospital treatment: validity and reliability of the German version of the post-discharge coping difficulty scale

**DOI:** 10.1186/s41687-024-00806-9

**Published:** 2024-11-05

**Authors:** Matthias Marsall, Thorsten Hornung, Alexander Bäuerle, Marianne E. Weiss, Martin Teufel, Matthias Weigl

**Affiliations:** 1 Institute for Patient Safety (IfPS), University Hospital Bonn, Bonn, Germany; 2 Medical Management Division, University Hospital Bonn, Bonn, Germany; 3 Clinic for Psychosomatic Medicine and Psychotherapy, LVR–University Hospital Essen, University of Duisburg-Essen, Essen, Germany; 4 Center for Translational Neuro- and Behavioral Sciences (C-TNBS), University of Duisburg-Essen, Essen, Germany; 5 College of Nursing, Marquette University, Milwaukee, WI USA

**Keywords:** Patient safety, Care transition, Psychometric properties, Patient survey, Measurement invariance, Patient-reported outcome measures

## Abstract

**Background:**

Patients transitioning between different care contexts are at increased risk of experiencing adverse events. In particular, being discharged to home after inpatient treatment involves significant risks. However, there is a lack of valid and internationally comparable assessment tools on patients’ experiences of difficulties following hospital discharge. Therefore, this study aimed to adapt and validate the German version of the post-discharge coping difficulty scale (PDCDS-G).

**Methods:**

Patients were recruited at a German university hospital. 815 adult patients participated in a self-report survey following an inpatient stay of at least three days. Factorial validity of the PDCDS-G was evaluated via factor analyses. Further, examination of measurement invariance was performed. To establish criterion validity, associations with patients’ self-reported health status and occurrence of patient safety were determined. Further, group differences regarding patient characteristics, hospitalization factors, and survey-related variables were examined.

**Results:**

Factorial validity of the PDCDS-G was confirmed by a two-factorial model with good model fit. Both factors showed good to excellent reliability. The two-factor model achieved measurement invariance across all patient characteristics, hospitalization factors, and survey-related variables. Significant relationships with patients’ health status and the occurrence of patient safety incidents corroborate criterion validity of the PDCDS-G. Differential associations of the two PDCDS-G factors regarding patient characteristics, hospitalization, and survey-related variables were found.

**Discussion:**

Construct and criterion validity, as well as the reliability of the PDCDS-G, were verified. Further, instrument’s measurement invariance was confirmed allowing use of the scale for the interpretation of group differences and comparisons between studies.

**Conclusions:**

The PDCDS-G provides a validated and comparable patient-reported outcomes measure for patient experiences after hospital discharge to home. The PDCDS-G can be used for patient surveys in quality or patient safety improvement in care transition processes.

**Supplementary Information:**

The online version contains supplementary material available at 10.1186/s41687-024-00806-9.

## Background

Transitions of patients between different care facilities or healthcare contexts are associated with potential harm due to deficiencies of information transfer or discontinuity of care [1, 2]. In particular, transfer from inpatient treatment to home is a critical patient transition regarding quality and safety and health outcomes [3, 4]. Patients returning home after discharge from inpatient treatment are often affected by complex challenges in their health and life situation, resulting in potential coping difficulties, e.g., regarding their medication, deterioration in life satisfaction and functional health status, as well as a higher vulnerability to secondary diseases [5–7]. As a result, unplanned hospital readmissions or patient harm, for example, due to medication complications, are significant healthcare problems with negative consequences for patients and relatives as well as economic sequelae for the healthcare system [8–11].

Patient engagement is one of the pillars of patient safety in the World Health Organization’s Patient Safety Action Plan 2020-2030 [12]. Involving patients in the investigation of challenges that occur after hospital discharge is crucial for providing patient-centered, safe care and ensuring a smooth transition to home [13]. Nevertheless, available studies examining the quality of care transition and associated results for patients at home often lack valid and comparable patient-centered outcome measures [14]. In particular, considering patient experiences in capturing care outcomes is imperative for studying transition processes and patient safety [15]. Understanding patients’ experiences is vital to improving outcomes of healthcare delivery, transitions, and, ultimately, the advancement of health services [13]. Consequently, there is an urgent need for valid and reliable patient-reported outcomes measures (PROMs) to actively involve patients in post-discharge evaluation and improvement of healthcare [16].

The post-discharge coping difficulty scale (PDCDS) was developed to assess difficulties perceived by patients after being discharged from a healthcare facility as well as their confidence in self-care for health needs [17]. The original PDCDS was a one-factorial instrument with good validity and reliability [17, 18]. To the best of our knowledge, this scale is the only instrument with which patients can be validly asked about their perceptions and experiences in dealing with their health situation at home after discharge from inpatient hospital treatment. This scale was adopted in different settings (e.g., for parents of hospitalized children and postpartum mothers) [19–21]. Post-discharge coping difficulties have been demonstrated to be valid outcomes for failures in discharge management [18, 22].

The various forms of PDCDS (for parents of hospitalized children or for postpartum mothers) have already been translated into several languages and used internationally [19, 23–25]. In German health care, neither care transitions nor their outcomes are well surveyed from patients’ perspective [26, 27]. A German-language version of the PDCDS is also still missing. This lack of valid measurement tools hampers opportunities for process improvement and the inclusion of patient experience in discharge processes, thereby limiting patient safety research in this area.

### Objectives

This study examined the validity and reliability of the German version of the post-discharge coping difficulty scale (PDCDS-G). Specifically, we aimed to.

1. scrutinize the factorial validity and reliability of the PDCDS-G,
2. test its measurement invariance regarding important patient characteristics, hospitalization factors, and survey-related variables,
3. examine its criterion validity through determining relationships with patient safety incidents (i.e., unplanned hospital readmissions and medication complications) and health-related outcomes (i.e., physical and mental health status),
4. report group differences regarding patient characteristics, hospitalization factors, and survey-related variables.

## Methods

### Study design and setting

We conducted a cross-sectional survey among discharged hospital patients. Participants were recruited between May and August 2022 at a large hospital in North Rhine-Westphalia, Germany. The academic medical center serves more than 500,000 patients annually in inpatient, outpatient, and emergency care settings. Eligible patients were identified via the hospital administration records. A total of 3945 patients were included and received a self-report survey via mail three weeks after their discharge from inpatient treatment. Participation was possible via the paper-pencil version of the survey or an equivalent web-based version (Unipark; Tivian XI GmbH), accessible via a web link printed in the paper-pencil questionnaire. If patients chose to participate by paper-pencil, they could return the completed questionnaire by mail free of charge. Further, patients were free to choose whether to complete the questionnaire themselves or have a relative complete it for them. Survey participation was anonymous and not compensated. The questionnaire included comprehensive study information, disclosure, and contact information.

The study was conducted according to the Declaration of Helsinki. The Ethics Committee of the Medical Faculty of the University of Bonn approved the study protocol (ref no. 107/22). The study protocol was registered at the German Clinical Trials Register under ref. DRKS00028947. Results are reported in accordance with the Strengthening the Reporting of Observational Studies in Epidemiology guidelines (STROBE) [28].

### Participants

We applied the following inclusion criteria: participants had to be at least 18 years old; they had to have been treated as inpatients with a minimal length of hospital stay of at least 3 days; they had to be discharged to home (with or without home care services) and not to any treatment facility or follow-up care like nursing homes or rehabilitation centers. Patients from all clinical departments and specialties (except pediatrics) were taken into account when sending out the questionnaires.

### Measurements

#### The German version of the post-discharge coping difficulty scale (PDCDS-G)

The assessment of coping difficulties after discharge was carried out with the PDCDS-G. This instrument is based on the original, English-language version of the PDCDS [17]. Previously, a German-language translation of the PDCDS for parents of hospitalized children (Ped-PDCDS) was adopted [29]. Since there were only slight differences in wordings between the PDCDS and the Ped-PDCDS, we adapted the items of the adult version of the questionnaire in consultation with the developer of the original items. Additionally, to strengthen content validity, we discussed each instrument item regarding comprehensibility and clarity with a nursing scientist with extensive practical experience. Further, to be more consistent to other self-report instruments, we adapted the items so that they were not questions, but statements. In line with this, we have changed the response options to an agreement scale from ‘Strongly Disagree’ to ‘Strongly Agree’. The response scale was set from 1 to 5. We then compiled a final German adult version of the PDCDS.

Consistent with its original, the PDCDS-G consists of 10 items covering different patient experiences of coping difficulties after hospital discharge. Response options were from 1 = ‘Strongly Disagree’ to 5 = ‘Strongly Agree’. Items eight to ten were reverse scored so that higher values indicate higher levels of coping difficulties. The English original items are freely available [19]. The items of the German adult version of the post-discharge coping difficulty scale are presented in Supplementary Material A.

#### Patient safety incidents and health-related outcomes after hospital discharge

Patients were further surveyed if they experienced the following patient safety incidents after being discharged to home: unplanned hospital readmissions and medication complications (such as incorrect dosage or unexpected side effects). Dichotomous response options (yes vs. no) were used. Both items were self-developed and utilized in a previous study [30].

We further assessed two health-related outcome items (i.e., physical health status and mental health status) to determine the criterion validity of the PDCDS-G. Both items were used in previous studies [31–33]. The items were answered on 11-point Likert scales, with higher scores indicating better health status after discharge.

#### Patient characteristics, hospitalization factors, and survey-related variables

Patients’ age (in years), gender (female, male, diverse), and the type of their health insurance (statutory health insurance, private health insurance, other health insurance) were assessed as patient characteristics. The following hospitalization factors were captured: length of hospital stay (in days), need for ICU treatment (yes vs. no), and the perceived timeliness of discharge (timely, too late, too early). Moreover, the survey inquired whether patients or their family members had answered it. We further recorded whether the questionnaires were completed as paper-pencil versions or via the online survey.

### Statistical analysis

We reported characteristics of the study sample as mean values and standard deviations as well as median and response range for continuous variables and as numbers and percentages for categorical variables. Item statistics of the PDCDS-G items were reported with mean values, standard deviations, skewness, and response distribution in percent.

The following analytical steps were used to determine the validity and reliability of the PDCDS-G:

Before establishing factorial validity through factor analyses, we randomly split the study sample into two subsamples of equal size [34]. The first half was used as a training sample for exploratory factor analysis (EFA) in the first analytical step. We used the Kaiser-Meyer-Olkin (KMO) and Bartlett’s test of sphericity to determine the appropriateness of the data for factor analyses. Maximum likelihood estimation with Promax rotation was used, and we assumed factor loadings higher than 0.4 to be significant [35]. We extracted factors based on the Kaiser criterion with Eigenvalue > 1 [35].

The second half of the sample was used for confirmatory factor analyses (CFA) and tests of measurement invariance. In this second step, we deployed the weighted least squares means and variance adjusted estimator (WLSMV) to consider that the PDCDS-G items were categorical indicator variables [36]. We examined two different factorial models: in model 1, all ten items were loading on a single factor (baseline: single factor model). Model 2 was based on the EFA-based model established in Step 1. We compared model fit indices, respectively, considering the following criteria: comparative fit index (CFI) and Tucker Lewis index (TLI) should be about 0.95 or higher, root mean square error of approximation (RMSEA) reported with 90% confidence interval (CI) should not exceed 0.06, and standardized root mean square residual (SRMR) should be below 0.08 [37]. Scale reliabilities were examined with McDonald’s Omega. The test and the training sample did not significantly differ regarding any of the patient characteristics, hospitalization factors, or survey-related variables.

Next, we tested the measurement invariance of the PDCDS-G regarding patient characteristics, hospitalization factors, and survey-related variables as a prerequisite for interpreting mean differences between groups of patients [38, 39]. For this purpose, the following data adjustments were performed: we performed median splits regarding patients’ age and length of hospital stay to obtain two group variables, respectively. Due to small subgroup sample sizes, we excluded patients identifying themselves as ‘diverse’ (*n*=3) and those who indicated their health insurance as ‘other’ (*n*=4). Regarding timeliness of discharge, indications ‘too late’ and ‘too early’ were combined as one group (‘not timely’) due to small samples. We tested three increasingly restrictive models via multigroup-CFA: first, by equally setting the number of factors and the pattern of factor-indicator relationships (configural invariance); second, by equally setting the factor loadings (metric invariance); and third, by equally setting the intercepts of indicators in (scalar invariance) between groups for each of the patient characteristic, hospitalization factor, and survey-related variable [40, 41]. To determine whether measurement invariance could be assumed, we tested the more restrictive model against the less restrictive model using the following cut-offs: changes in CFI (ΔCFI), which should not be greater than −0.01, and changes in RMSEA (ΔRMSEA), which should not be greater than 0.015 [42].

Further, Pearson and point biserial correlation analyses were deployed to determine the relationship between the PDCDS-G and patient safety incidents and health-related outcomes.

In the last step, we used Pearson correlation analyses and t-tests to examine whether the PDCDS-G differed concerning the patient characteristics, hospitalization factors, and survey-related variables. The significance level was set to *p* = 0.05. Cohens d was reported as effect size for t-tests. All cases with more than 30% missing answers were excluded from the analyses. Listwise deletion was applied for remaining missing values. All analyses were carried out using R and RStudio [43, 44].

## Results

### Participants and study sample description

Eight hundred and fifteen patients eligible responded to our survey (response rate: 20.7%). We excluded 40 participants from the analyses due to excessive missing responses of more than 30%. The final sample consisted of 775 patients. On average, patients were 60.5 years old (SD=18.0, Median=63, range from 19 to 94). The average length of hospital stay was 10.1 days (SD=16.4, Median=5, range from 3 to 165). An overview of the categorical study sample characteristics is shown in Table 1.

**Table 1:** Study sample characteristics

Characteristic	*n* (%)	Missing *n* (%)
**Gender**		6 (0.8%)
Female	377 (48.6)	
Male	389 (50.2)	
Diverse	3 (0.4)	
**Health Insurance**		12 (1.5%)
Statutory health insurance	596 (76.9)	
Private health insurance	163 (21.0)	
Other health insurance	4 (0.5)	
**ICU care**		30 (3.9%)
No	545 (70.3)	
Yes	200 (25.8)	
**Perceived timeliness of discharge**		28 (3.6%)
Timely	622 (80.3)	
Too early	109 (14.1)	
Too late	16 (2.1)	
**Respondent**		81 (10.5%)
Patient	615 (79.4)	
Relative	79 (10.2)	
**Survey type**		–
Paper-pencil survey	644 (83.1)	
Online survey	131 (16.9)	

### Item statistics and factorial validity of the PDCDS-G

Except for item 1, all items of the PDCDS-G were positively skewed. The item means ranged from 1.72 to 3.36. The proportion of missing values was between 0.5 and 2.3%. Detailed statistics of all items are presented in Supplementary Material A.

#### Exploratory factor analysis

The KMO value was 0.88, indicating that the PDCDS-G items were suitable for factor analysis. This finding was supported by a significant Bartlett’s test of sphericity (Chi² = 2406.97, df = 45, *p* < 0.001). Exploratory factor extraction revealed a two-factor solution accounting for 61% of the variance. No significant cross-loading of items between the two factors were identified. The factor loadings are shown in Table 2.

**Table 2:** Factor loadings of the PDCDS items (EFA)

Item #	Factor 1	Factor 2
1	**0.72**	−0.06
2	**0.78**	−0.04
3	**0.85**	0.06
4	**0.68**	0.18
5	**0.79**	−0.03
6	**0.81**	0.03
7	**0.68**	−0.02
8	0.06	**0.76**
9	−0.12	**0.98**
10	0.10	**0.60**

*Notes N* = 388; significant factor loadings are in bold

#### Confirmatory factor analysis

The empirically established EFA model consisted of two interrelated factors (cf., Table 2). Therefore, this two-factor solution was used in the following CFA. Table 3 summarizes the results for model fits.

**Table 3:** Results of the confirmatory factor analyses

Model	Chi²	df	CFI	TLI	RMSEA	RMSEA CI	SRMR
Single-factor model	127.107	35	0.977	0.970	0.097	0.086–0.110	0.098
Two-factor model	44.096	34	0.997	0.997	0.055	0.045–0.065	0.050

*Notes N*=350. Estimator: WLSMV; df: degrees of freedom; CFI: comparative fit index; TLI: Tucker Lewis index; RMSEA: root mean square error of approximation; CI: 90% confidence interval; SRMR: standardized root mean square residual

Results revealed that the two-factor model fits the data better than the single-factor model. The difference in the Chi² values between the two models was highly significant (*p*<0.001), underpinning that the two-factor model was superior to the single-factor model. All criteria for an excellent model fit were met by the two-factor model. All item factor loadings were significant and ranged from 0.69 to 0.89. The two factors were significantly interrelated (*r*=0.59, *p*<0.001).

This observed model indicated the separation of the PDCDS-G into two factors: factor 1 describes the occurrence of specific difficulties after discharge (i.e., PDCDS-G-difficulties), while factor 2 describes the extent to which patients feel they can cope at home after discharge (i.e., PDCDS-G-coping). Both factors showed high reliabilities (McDonald’s Omega of 0.92 and 0.84, respectively). PDCDS-G-difficulties had a mean of M = 2.71 (SD = 1.13); PDCDS-G-coping had a mean of M = 2.08 (SD = 1.03). The overall mean of all items was M = 2.52 (SD = 0.99) with reliability of 0.93.

The two-factor model and the two subscales were used for subsequent analyses.

### Measurement invariance of the PDCDS-G

Measurement invariance analyses revealed that, regarding all sociodemographic, care-related, and survey-related characteristics, none of the changes in CFI or RMSEA exceeded the criteria for confirmation of measurement invariance. Therefore, strong measurement invariance of the PDCDS-G could be assumed. The detailed results of the analyses are presented in Supplementary Material B.

### Criterion validity of the PDCDS-G

All following analyses were deployed with the whole study sample. To determine the criterion validity of the PDCDS-G, we performed correlation analyses of the two subscales with patient safety incidents and health-related outcomes. 65 (8.4%) and 85 (11.0%) of patients reported unplanned hospital readmissions and medication complications, respectively. 18 (2.3%) reported to have experienced both patient safety incidents. The average self-rated physical health was M = 6.9 (SD = 2.4) and the average self-rated mental health was M = 7.6 (SD = 2.7). The results regarding the relationships of the PDCDS-G with patient safety incidents and health-related outcomes are shown in Table 4. We observed significant relationships of both PDCDS-G subscales with the two four outcomes, respectively. The correlations of PDCDS-G were low for patient safety incidents and moderate-to-high for health outcomes. These results underpinned the criterion validity of the PDCDS-G.

**Table 4:** Relationships of PDCDS-G with patient safety incidents and health-related outcomes

Characteristic	PDCDS-G-difficulties	PDCDS-G-coping
	Correlation coefficient	
**Patient safety incidents**		
Unplanned hospital readmission	*r* = 0.22***	*r* = 0.15***
Medication complications	*r* = 0.28***	*r* = 0.24***
**Health-related outcomes**		
Physical health	*r* = −0.49***	*r* = −0.34***
Mental health	*r* = −0.52***	*r* = −0.36***

*Notes* ***p<0.001

### Relationships of PDCDS-G with patients, hospitalization factors, and survey variables

We examined relationships between the two PDCDS-G factors and patient characteristics, hospitalization factors, and survey-related variables. The results of these analyses are summarized in Table 5.

**Table 5:** Relationships of PDCDS-G with patient, hospitalization, and survey variables

Characteristic	PDCDS-G-difficulties			PDCDS-G-coping		
	Mean	SD	Significance test/effect size (d)	Mean	SD	Significance test/effect size (d)
**Patient characteristics**						
Age	–	–	*r* = 0.10, *p* < 0.01	–	–	*r* = 0.06, *p* = 0.09
Gender^a^			*t* = 1.45 (764), *p* = 0.15; d = 0.10			*t* = 0.63 (764), *p* = 0.53; d = 0.05
Female	2.77	1.15		2.10	1.07	
Male	2.65	1.11		2.05	1.00	
**Health insurance** ^**b**^			*t* = −3.71 (757), *p* < 0.001; d = 0.34			*t* = −2.64(757), *p* < 0.01; d = 0.24
Statutory health insurance	2.79	1.14		2.14	1.05	
Private health insurance	2.43	1.05		1.90	0.96	
**Hospitalization factors**						
Length of hospital stay	–	–	*r* = 0.13, *p* < 0.001	–	–	*r* = 0.12, *p* < 0.001
ICU care			*t* = −4.97 (743), *p* < 0.001; d = 0.40			*t* = −3.32 (743), *p* < 0.001; d = 0.26
No	2.59	1.10		2.02	0.98	
Yes	3.05	1.18		2.30	1.16	
**Timely discharged** ^c^			*t* = −7.59 (745), *p* < 0.001; d = 0.71			*t* = −6.97(745), *p* < 0.001; d=0.63
Timely	2.57	1.06		1.96	0.95	
Not timely	3.37	1.21		2.64	1.21	
**Survey-related variables**						
Respondent			*t* = −9.33 (692), *p* < 0.001; d=1.13			*t* = −10.45 (692), *p* < 0.001; d=1.18
Patient	2.57	1.07		1.94	0.95	
Relative	3.76	1.04		3.14	1.10	
Survey type			*t* = 2.08(773), *p* < 0.05; d=0.20			t=1.49 (773), *p* = 0.14; d=0.15
Paper-pencil	2.75	1.14		2.10	1.04	
Online	2.53	1.10		1.95	0.99	

*Notes*^a^Patients self-identifying as diverse (*n*=3) were treated as missings. ^b^Patients indicated health insurance as other (*n*=4) were not included. ^C^Indications ‘too late’ (*n*=16) and ‘too early’ (*n*=109) were merged as ‘not timely’. Significant relationships are in bold

Regarding patients’ age and survey type, results showed differential relationships with the two subscales. While higher age was related to higher perceived difficulties, there was no relationship with perceived coping abilities. Similarly, patients who answered the survey via paper-pencil version (vs. online version) reported higher perceived difficulties but no difference regarding coping abilities. However, patients who used the online version of the survey were significantly younger than those who used the paper-pencil version (M=51.6 vs. M=62.3, respectively; t_755_=6.32, *p*<0.001). More difficulties and lower coping abilities were reported by patients with statutory health insurance (vs. private health insurance), by those who needed intensive medical care, were not timely discharged, and where relatives completed the questionnaire. Patients’ gender was not related to any of the subscales.

## Discussion

### Principal findings

With this study, we provide a valid and reliable German version of the post-discharge coping difficulty scale as an important tool for the practical application and further research of self-reported difficulties of patients after discharge from inpatient hospital treatment. The valid assessment of patient-reported outcomes measures after hospital inpatient care is essential for including patients’ experiences in healthcare and therefor advancing patient safety in care transitions [15, 16]. After hospitalization, many patients experience difficulties in dealing with their health and healthcare needs [7, 18, 45–47]. However, a systematic and comparable assessment of patients’ coping difficulties after hospital discharge was lacking. Therefore, this study aimed to validate the PDCDS-G and thus offer research and practical application of a tool that can be used to strengthen patient engagement in the discharge process. Drawing upon a large sample of patients after hospital discharge, we confirmed the scientific utility of this new tool to survey post-discharge difficulties among German-speaking patients.

With regard to the first research objective, our findings provide evidence for the validity and reliability of the German adult version of the post-discharge coping difficulty scale. We finally extracted two factors and confirmed the structural validity of this two-factorial solution. However, this two-factorial model is inconsistent with the original single-factor solution [17, 18]. Our proposed model of two subscales can be interpreted as ‘experiences of difficulties’ and ‘experience of coping abilities’ after hospital discharge. These two factors represent a content-valid representation of the items assigned to the respective factors. The assumption of a two-factorial structure is supported by partly differential relationships of the two factors with the health-related outcomes, patient characteristics, hospitalization factors, and survey-related variables discussed further below. Additionally, different factor structures of an instrument in different languages and countries may reflect different characteristics of the healthcare system and the patients it cares for [48]. Concerning the reliability of the PDCDS-G, both factors achieved good to excellent internal consistency.

Our study’s second objective was to examine the measurement invariance of the PDCDS-G regarding various sociodemographic, care-related, and survey-related characteristics. Measurement invariance is essential for comparing different studies with different study settings and indicates that an instrument can represent group differences [38–40]. Our analyses revealed strong measurement invariance of the PDCDS-G. This result is highly relevant, as it indicates that the PDCDS-G can be used independently of sociodemographic, care-related, and survey-related characteristics and that potential group differences may be interpreted as such. Additionally, our results strongly supported invariance for the method of PDCDS-G completion. More precisely, the results were independent of whether patients filled it out by paper-pencil or via the online version, as well as independent of whether it was filled out by patients themselves or their relatives on their behalf. This finding confirms that PDCDS-G can be provided in different modes and that the results of different studies can be validly compared.

To address the third study aim, we also investigated its relationships with patient safety incidents and health-related outcomes. Criterion validity of the PDCDS-G was consistently confirmed via significant correlations of PDCDS-G with the occurrence of patient safety incidents and experienced levels of health-related outcomes. These results corroborate previous findings that patients who perceive post-discharge difficulties were more likely to experience adverse events and poorer physical and mental health [18, 49]. Taken together, these results substantiate the criterion validity of the PDCDS-G.

Regarding the fourth study aim, we reported group differences regarding patient characteristics, hospitalization factors, and survey-related variables. Concerning individual characteristics, patient’s age was significantly related to the perception of more post-discharge difficulties but not the perceived coping abilities. Even though the relationship was moderate, this points out that older patients may experience more post-discharge difficulties but are equally capable of coping with difficulties as younger patients. However, this result is inconsistent with previous results and should be examined in more detail in further studies [22]. Interestingly, our results showed that patients with statutory health insurance experienced more post-discharge difficulties and less ability to cope with difficulties than patients with private insurance. However, it has been shown that patients with private health insurance have better health-related outcomes than patients with statutory health insurance [50]. Concerning hospitalization factors, our results demonstrate that higher post-discharge coping difficulties were experienced in patients with extended hospital stays who needed ICU care and perceived that they were not discharged in a timely manner. Although this is not entirely consistent with previous findings regarding the PDCDS [22], these results consistently show that patients with a high level of stress during hospitalization experience significant difficulties in dealing with their health even after discharge. Further, it should be emphasized that these results align with previous findings indicating that hospitalization factors like the length of hospital stay and the need for intensive medical care are risk factors for experiencing post-discharge difficulties and complications [51]. The use of the paper-pencil version of the PDCDS-G was related with the experience of more health difficulties. However, as paper-pencil users were significantly older than users of the online survey, we assume that this relationship could be determined by patients’ age.

The PDCDS-G is a valid and reliable instrument for surveying patient experiences after inpatient hospital treatment. This analysis is an essential step toward engaging patients in health care and gaining a better understanding of discharge processes and ensuing patient-reported outcomes. For clinical practice, the PDCDS-G is an instrument for assessing and following up on patient experiences after inpatient treatment. It can also be used as a screening tool to identify patients with special care needs or risk groups that need particular attention after hospital discharge. As the PDCDS-G captures fundamental ‘experiences with difficulties’ and ‘experience of coping abilities’, the instrument is well suited for broad use across different patient cohorts. However, specific coping requirements due to specific primary illnesses should be assessed using specialized instruments (e.g., the Cancer Coping Questionnaire [52], the Presbyopia Impact and Coping Questionnaire [53], the Voice Disability Coping Questionnaire [54]). Because of the high correlation between the subscales of the PDCDS-G, both the subscales and the overall scale can be utilized as valid instruments. However, we recommend that both subscales are evaluated separately, as they reflect different experiences of the patients. Due to its brevity, the instrument is easy to implement in clinical practice as well as scientific research. As the instrument focusses on coping difficulties shortly after hospital discharge, the instrument should be used shortly after discharge. Previous research has shown that communication and patient involvement are key aspects of smooth and safe patient transitions [30, 55]. Future research should therefore investigate the potential influence of the transition process on patients’ perception of difficulties and coping abilities after their discharge to home.

### Limitations

Although we applied a rigorous testing procedure and used a large sample of patients across various medical specialties, our results should be interpreted in the light of some limitations. First, we used a convenience sampling approach, and participation was voluntary. Thus, sampling bias cannot be excluded. The generalizability of our results may be limited as the study sample included patients of one German University Hospital and with a large proportion of older patients. Due to the relatively high age of the participants of this study, potential response burden could be experienced, which could lead to misunderstandings of the items and incorrect responses. Further, there was a large variance in the reported length of hospital stay. However, patients with various diseases from different clinical departments were studied and underwent a broad spectrum of clinical procedures and medical interventions strengthening generalizability. By applying a cross-sectional study design, we showed correlations. However, our study was not designed to interpret causal relationships between the PDCDS-G and patient safety incidents, health-related outcomes as well as patient characteristics, hospitalization factors, and survey-related variables. Also, the study design did not allow to carry out further reliability measurements, such as test-retest-reliability. When testing the measurement invariance of the timeliness of discharge, groups “too late” and “too early” were combined due to the group sizes. However, this may have introduced an imprecision in the analysis, as the individuals in the two groups may have experienced different coping difficulties.

## Conclusion

Our results demonstrate that the PDCDS-G is a valid and reliable scale to assess post-discharge coping difficulties from the patients’ perspective. The instrument is measurement invariant and can be used independently from significant patient sociodemographic, care-related, and survey-related characteristics. Criterion validity of the PDCDS-G was confirmed. Further, the instrument is significantly related to several sociodemographic, care-related, and survey-related characteristics. It can be used for German-speaking patient samples to explore and identify patient experiences and improvement needs in the course of care after hospital discharge.

## Electronic supplementary material

Below is the link to the electronic supplementary material.

Supplementary Material 1

## Data Availability

The datasets used and/or analyzed during the current study are available from the corresponding author upon reasonable request.
