# Supplementary material for: Coping difficulties after inpatient hospital treatment: validity and reliability of the German version of the post-discharge coping difficulty scale
Source: J Patient Rep Outcomes. 2024 Nov 5;8:125. doi: 10.1186/s41687-024-00806-9 (PMC11538096; doi:10.1186/s41687-024-00806-9)
Supplement: Supplementary file 1 — Supplementary Material 1 [file 41687_2024_806_MOESM1_ESM.docx]

# Supplementary material

## Supplementary material A. Item wording and statistics of the German adult version of the post-discharge coping difficulty scale

| **#** | **Item** | **Mean** | **SD** | **Skew** | **Response distribution in percentage^a^** | | | | | |
| --- | --- | --- | --- | --- | --- | --- | --- | --- | --- | --- |
|  |  |  |  |  | **1** | **2** | **3** | **4** | **5** | **mis.** |
| 1 | Mein Leben war inzwischen anstrengend. | 3.36 | 1.34 | -0.37 | 12.1 | 16.5 | 18.3 | 27.6 | 24.6 | 0.8 |
| 2 | Ich hatte viele Schwierigkeiten dabei, mich zu Hause zu erholen. | 2.59 | 1.35 | 0.41 | 25.5 | 29.2 | 14.3 | 17.5 | 11.4 | 2.1 |
| 3 | Ich hatte viele Schwierigkeiten dabei, mich selbst zu versorgen. | 2.38 | 1.38 | 0.62 | 34.8 | 26.2 | 11.7 | 14.7 | 10.5 | 2.1 |
| 4 | Ich hatte viele Schwierigkeiten dabei, meine medizinische Versorgung zu übernehmen. | 2.22 | 1.33 | 0.81 | 40.3 | 25.8 | 12.0 | 11.9 | 8.8 | 1.3 |
| 5 | Für meine Familie/näheren Bezugspersonen war es eine schwierige Zeit. | 2.85 | 1.47 | 0.10 | 25.5 | 20.6 | 11.7 | 22.6 | 17.2 | 2.3 |
| 6 | Ich habe viel Hilfe benötigt, um mich selbst zu versorgen. | 2.62 | 1.47 | 0.36 | 31.6 | 22.7 | 11.5 | 17.7 | 15.4 | 1.2 |
| 7 | Ich habe viel emotionale Unterstützung benötigt. | 2.89 | 1.47 | 0.01 | 26.2 | 17.8 | 12.1 | 26.1 | 16.8 | 1.0 |
| 8 | Ich habe mich sicher gefühlt, für meine eigenen Bedürfnisse sorgen zu können. (recoded) | 2.42 | 1.28 | 0.62 | 27.9 | 32.9 | 14.1 | 14.5 | 8.6 | 2.1 |
| 9 | Ich war gut in der Lage, mich um meine medizinische und medikamentöse Behandlung zu kümmern. (recoded) | 2.11 | 1.27 | 1.09 | 40.0 | 34.7 | 7.1 | 8.8 | 8.6 | 0.8 |
| 10 | Ich habe mich nach dem Klinikaufenthalt gut wieder zu Hause eingelebt. (recoded) | 1.72 | 1.04 | 1.66 | 55.5 | 29.0 | 6.5 | 4.8 | 3.7 | 0.5 |

Items 8, 9, and 10 were reverse scored. ^a^mis.=missing; response options English: 1 = ‘Strongly Disagree’ - 5 = ‘Strongly Agree’; Response options German: 1 = ‘Stimme überhaupt nicht zu’ - 5 = ‚ Stimme voll und ganz zu ‘.

## Supplementary material B. Measurement invariance of the PDCDS-G regarding sociodemographic, care-related, and survey-related characteristics

| **Characteristic** | **Model** | **Chi²** | **df** | **CFI** | **ΔCFI** | **RMSEA** | **ΔRMSEA** |
| --- | --- | --- | --- | --- | --- | --- | --- |
| **Reference CFA model** | |  |  | **0.997** | **-** | **0.055** | **-** |
| Age ^a^ | configural | 167.0 | 68 | 1.000 | 0.003 | 0.058 | 0.003 |
|  | metric | 147.4 | 76 | 1.000 | 0.000 | 0.058 | 0.000 |
|  | scalar | 163.0 | 84 | 1.000 | 0.000 | 0.058 | 0.000 |
| Gender ^b^ | configural | 153.1 | 68 | 1.000 | 0.003 | 0.053 | -0.002 |
|  | metric | 132.6 | 76 | 1.000 | 0.000 | 0.051 | -0.002 |
|  | scalar | 153.9 | 84 | 1.000 | 0.000 | 0.054 | 0.003 |
| Health Insurance ^c^ | configural | 153.2 | 68 | 1.000 | 0.003 | 0.055 | 0.001 |
|  | metric | 122.1 | 76 | 1.000 | 0.000 | 0.049 | -0.006 |
|  | scalar | 128.8 | 84 | 1.000 | 0.000 | 0.047 | -0.002 |
| ICU care | configural | 142.1 | 68 | 1.000 | 0.003 | 0.053 | -0.001 |
|  | metric | 136.0 | 76 | 1.000 | 0.000 | 0.054 | 0.001 |
|  | scalar | 141.0 | 84 | 1.000 | 0.000 | 0.050 | -0.004 |
| Timely discharged ^d^ | configural | 148.0 | 68 | 1.000 | 0.003 | 0.056 | 0.002 |
|  | metric | 139.8 | 76 | 1.000 | 0.000 | 0.059 | 0.003 |
|  | scalar | 143.5 | 84 | 1.000 | 0.000 | 0.055 | -0.004 |
| Length of stay ^a^ | configural | 178.3 | 68 | 0.989 | -0.008 | 0.060 | 0.005 |
|  | metric | 130.7 | 76 | 0.991 | 0.002 | 0.051 | -0.009 |
|  | scalar | 145.7 | 84 | 0.990 | -0.001 | 0.052 | 0.001 |
| Respondent | configural | 147.4 | 68 | 0.988 | -0.010 | 0.060 | 0.005 |
|  | metric | 138.5 | 76 | 0.986 | -0.001 | 0.059 | 0.000 |
|  | scalar | 167.1 | 84 | 0.981 | -0.005 | 0.067 | 0.008 |
| Survey type | configural | 163.1 | 68 | 0.990 | -0.007 | 0.056 | 0.002 |
|  | metric | 150.8 | 76 | 0.988 | -0.002 | 0.059 | 0.003 |
|  | scalar | 157.0 | 84 | 0.988 | 0.000 | 0.056 | -0.003 |

Notes. ^a^ Sample was split into two groups via median split. ^b^ Patients who identified themselves as diverse (n=3) were excluded. ^c^ Patients indicated health insurance as other (n=4) were not included. ^d^ Indications ‘too late’ (n=16) and ‘too early’ (n=109) were combined as one group.
